# Effect of streptozotocin-induced diabetes on myocardial blood flow reserve assessed by myocardial contrast echocardiography in rats

**DOI:** 10.1186/1475-2840-7-26

**Published:** 2008-09-02

**Authors:** Bernard Cosyns, Steven Droogmans, Sophie Hernot, Céline Degaillier, Christian Garbar, Caroline Weytjens, Bram Roosens, Danny Schoors, Tony Lahoutte, Philippe R Franken, Guy Van Camp

**Affiliations:** 1 Cardiology department, UZ Brussel, VUB, Brussels, Belgium; 2 Nuclear Medicine department, UZ Brussel, VUB Brussels, Belgium; 3 In Vivo Cellular and Molecular Imaging, VUB, Brussels, Belgium; 4 Department of Pathology, UZ Brussel, VUB, Brussels, Belgium

## Abstract

The role of structural and functional abnormalities of small vessels in diabetes cardiomyopathy remains unclear. Myocardial contrast echocardiography allows the quantification of myocardial blood flow at rest and during dipyridamole infusion. The aim of the study was to determine the myocardial blood flow reserve in normal rats compared with Streptozotocin-induced diabetic rats using contrast echocardiography.

We prospectively studied 40 Wistar rats. Diabetes was induced by intravenous streptozotocin in 20 rats. All rats underwent baseline and stress (dipyridamole: 20 mg/kg) high power intermittent imaging in short axis view under anaesthesia baseline and after six months. Myocardial blood flow was determined and compared at rest and after dipyridamole in both populations. The myocardial blood flow reserve was calculated and compared in the 2 groups. Parameters of left ventricular function were determined from the M-mode tracings and histological examination was performed in all rats at the end of the study.

At six months, myocardial blood flow reserve was significantly lower in diabetic rats compared to controls (3.09 ± 0.98 vs. 1.28 ± 0.67 ml min-1 g-1; p < 0.05). There were also a significant decrease in left ventricular function and a decreased capillary surface area and diameter at histology in the diabetic group.

In this animal study, diabetes induced a functional alteration of the coronary microcirculation, as demonstrated by contrast echocardiography, a decrease in capillary density and of the cardiac systolic function. These findings may offer new insights into the underlying mechanisms of diabetes cardiomyopathy.

## Introduction

Diabetic cardiomyopathy is known to develop in humans in the absence of coronary or hypertensive disease [1]. The mechanism by which diabetic cardiomyopathy develops has been studied in literature using in vivo and ex vivo experiments [2,3]. It has been postulated that endothelial dysfunction, endomyocardial fibrosis, direct toxic effect of hyperglycemia on cardiomyocytes and autonomic neuropathy play an important role. Although recent studies have suggested abnormalities in coronary small vessels in humans [4,5], similar to dilated cardiomyopathy [6,7], and in spontaneously diabetic rats [8], the association between vascular and myocardial disease in diabetes remains controversial.

Myocardial contrast echocardiography (MCE) has been shown to be a useful tool to estimate the myocardial blood flow (MBF) at rest and during stress in dogs and humans [9-13]. Echocardiography has been recently adapted to image hearts of rodents [14] and MCE has shown a good correlation of MBF estimates with microspheres technique [15].

In order to better investigate the role of functional vessel abnormalities and its relationship with structural small vessel abnormalities, we sought to apply this new MCE method in a small animal model of Streptozotocin-induced diabetes, to determine the coronary vascular reserve compared to normal controls, combined with histopathology. Furthermore, we aimed to establish a relationship between these alterations and the cardiac function.

## Methods

### Animals handling and study protocol

A total of 40 adult male Wistar Unilever rats (Harlan, The Netherlands) (10 weeks-old, 328 ± 7 g) were studied.

Diabetes mellitus was induced in 20 rats by a single intravenous injection of 45 mg/kg Streptozotocin (STZ) in a 0.1 mol/l citrate buffer solution. Three days after treatment with STZ, tail vein blood glucose samples were measured with Onetouch^R ^glucometer (Johnson & Johnson) to ensure induction of diabetes. During the entire study the animals were housed in stainless steel cages with sawdust bedding. They were kept at an average room temperature of 24°C, a relative humidity of 50% and a 12-hour day/night cycle. All rats had unlimited access to food and water during follow up.

MCE was performed at 1 and 6 months, both at rest and after dipyridamole (DIP) infusion (20 mg/kg over 10 minutes). Blood pressure was measured with the advanced auto-inflate blood pressure monitor with infrared sensor and tail-cuff from Harvard Apparatus^® ^France. The rats were anesthetized with pentobarbital 50 mg/kg intraperitoneally and allowing spontaneous respiration.

This study was approved by the Animal Research Committee at the Vrije Universiteit Brussel and conformed to the Institute for Laboratory Animal Research Guide for Care and Use of Laboratory Animals.

### Myocardial Contrast Echocardiography

The anterior chest hair was removed with a shaver and the rats were positioned in left lateral decubitus on a wooden bench. Recordings were made under continuous ECG monitoring by fixing electrodes on the paws. A 24-gauge cannula was inserted into a tail vein for infusion. The commercially available contrast agent Sonovue^® ^(Bracco Diagnostics, Inc.) is an aqueous suspension of stabilized SF_6 _microbubbles. The size of these microbubbles is between 1 and 10 μm, and their number is between 2 and 5 × 10^8 ^per mL. The solution was prepared according to the manufacturer's instructions. Subsequently this solution was diluted twice by adding 5 ml of sodium chloride 0.9% (same carrier fluid). A continuous infusion of the diluted solution of Sonovue^® ^was given using a dedicated pump (Bracco, Italy) at a rate of 0.3–0.4 ml/min.

**Figure 1:**
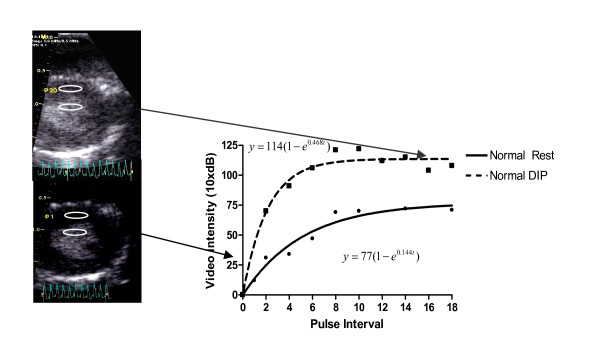
**Example of quantitative perfusion analysis. **The rBV was quantitatively estimated by the division of myocardial plateau video intensity and the adjacent left ventricular intensity in the near wall cavity at rest and during hyperaemia (DIP).

We used a Vivid 7 (GE, VingMed, Horton, Norway) with a 10 MHz (10S) probe. High power intermittent images were recorded in a parasternal short axis view at a depth of 2 cm at the level of the papillary muscles. The transmit power was set at a maximum (0 dB), with a mechanical index of 0.8. Gain settings were optimized at the beginning of the study and subsequently held constant. The pulsing interval (PI) was gated to the ECG (end systole) and progressively increased from 1 to 20 (depending on the heart rate). Images were digitally stored and measurements were analysed offline by the cardiologist, using the EchoPAC software (GE Vingmed, version 3.1.3). Regions of interest were placed on the anterior myocardium with a constant size (height: 1 mm, wide: 3 mm). The myocardial ROIs were individually adjusted by hand to carefully avoid the right and LV cavities. Videointensity (VI) was measured in these ROI's. As shown in the Figure 1, PI versus background-substracted images VI plots were then generated, and they were fitted to the exponential function y = A(1-e^-βt^), in which beta represents the rate of rise in signal intensity, which reflects microbubble velocity, and A is the peak plateau amplitude, which reflects the microvascular cross-sectional area or myocardial blood volume, as previously described [16]. Regional differences in VI due to concentration of microbubbles in blood may occur and the same concentration may not be achievable both at rest and during stress even with continuous infusions. Moreover, significant heterogeneity of acoustic energy in different parts of the scan sector makes comparisons of backscatter signals from segments at the margins or lower depths difficult with those in the centre and attenuation also results in spurious decreases in myocardial VI. Therefore, we quantitatively estimated the relative blood volume rBV by the division of myocardial plateau video intensity A and the adjacent left ventricular intensity in the near wall cavity (ALV), as described previously [12]. In accordance with Positron Emisson Tomography, at was set to 1.05 g·ml-1, and MBF was calculated with the following equation: MBF = rBV × β/ρT = (A-ALV) × β/ρT

Finally, the vasodilator reserve for beta and rBV, MBF, were calculated as the ratio of hyperaemic to baseline values for these parameters.

### Echocardiographic measurements

Grey scale images were recorded in a parasternal short axis view at a depth of 1.5 to 2 cm, using a linear 13 MHz probe (i13L), both at rest and after dypiridamole infusion. Left ventricular chamber diameter in end-systole (LVESD) and end-diastole (LVEDD), left ventricular anterior wall thickness in end-systole and end-diastole, and left ventricular posterior wall thickness in end-systole and end-diastole, as well as left ventricular fractional shortening (FS% = [(LVEDD – LVESD)/LVEDD] × 100), were determined from the M-mode tracings in a short axis view (average of three consecutive cycles) at the level of the papillary muscles.

### Histology

**Figure 2:**
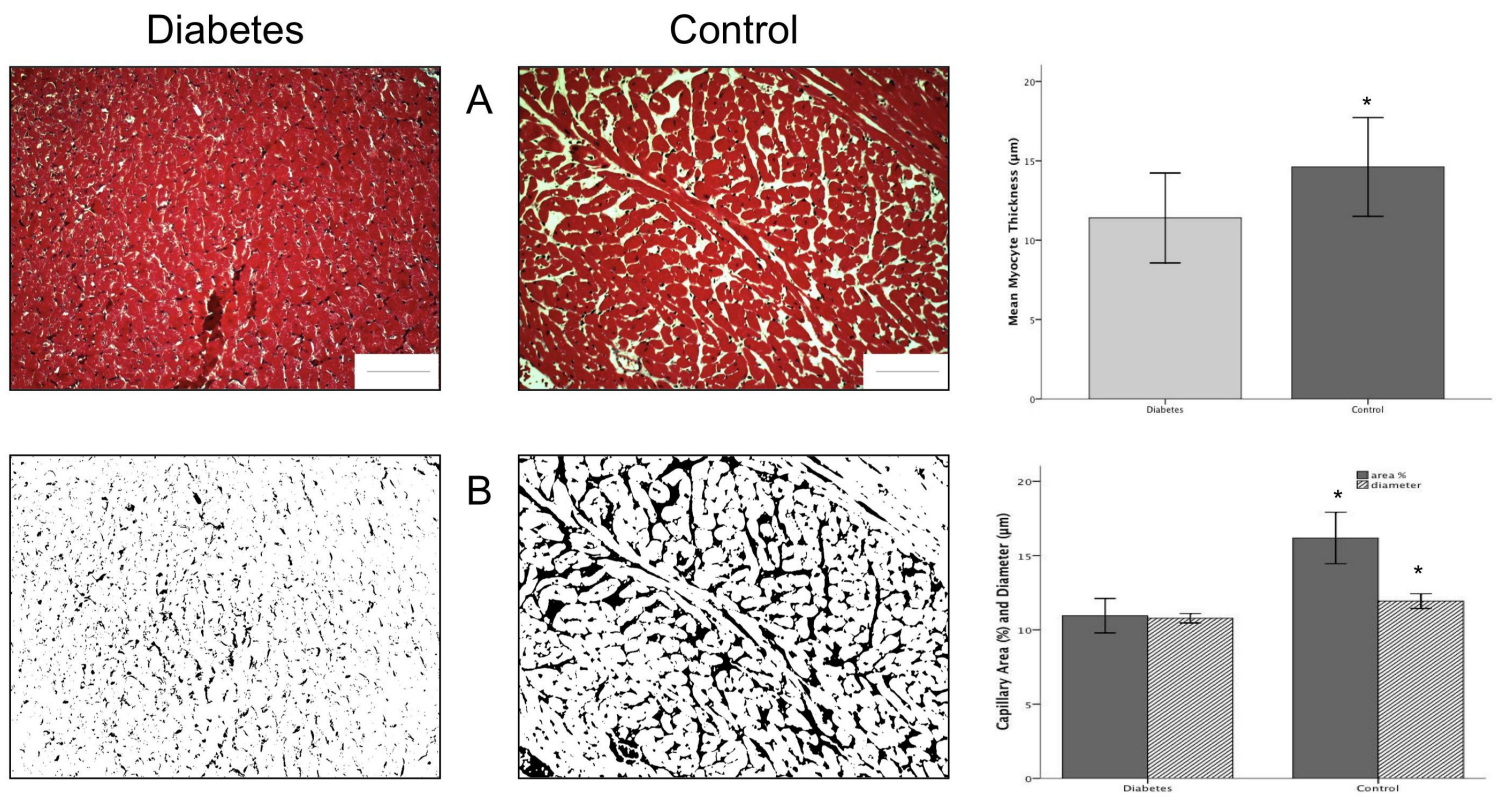
**Histological characteristics and analysis of the myocardium in the diabetic and control rats after 20 weeks.** Top panel (A), Masson's trichrome staining showed no evidence of myocardial fibrosis in both groups. Cardiomyocytes were smaller in the diabetic animals (upper right). Lower panel (B), the histological photographs (A) were subsequently converted to 8-bit grey scale images (not shown) and an appropriate threshold was chosen in order to visualize the capillaries only (black and white images, B). The percentage capillary area and capillary diameters were lower in the diabetic rats compared to controls (lower right). Magnification at 400× (A and B), scale bar is 100 μm, *P < 0.05.

At the end of the follow-up, all rats were killed with with 2 ml/kg sodium pentobarbital (CEVA, Brussels, Belgium) intravenously for histological studies. The hearts were immediately removed and fixed in 4% neutral buffered formalin for 2 hours. Three short-axis slices of myocardial tissue were obtained and embedded in paraffin. Heamatoxylin-eosin and Masson's trichrome staining were performed. Morphometry was performed by digital image analysis using a PC digital image camera (Digital Sight DS-5M, Nikon Corp, Japan) mounted on an Axiolab Zeiss light microscope (Carl Zeiss Corp, Germany) with a 10× objective (Acroplan, Zeiss). We used the NIH Image program (Image-J 1.35d, Nation Institutes of Health, Bethesda, USA). The program was calibrated with a graduated slide. Five randomly selected Masson's Trichrome microscopic images were used to evaluate the cardiomyocytes, interstitium and capillaries. The width of at least 10 left ventricle cardiomyocytes was measured in each section. Colour segmentation was applied to calculate the percentage of interstitial fibrosis. For the measurement of the capillary surface the histological photomicrographs were converted to 8-bit grey scale images. Subsequently, the threshold was adjusted until only the space between the cardiomyocytes was highlighted. This space was considered as a marker for capillary area. The percentage of black colored area was then measured. (Figure 2) Capillary diameters were measured manually. Microangiopathy involving arterioles, capillaries, and venules, and hyaline arteriosclerosis were also evaluated. An experienced pathologist performed the analyses blindly.

### Statistical analysis

Data are expressed as mean ± SD. The unpaired Student's *t *test was used for continuous variables to assess differences between groups, whereas ordinal variables were analyzed with the chi-square test. A subset of 10 rats underwent MCE three days after the first examination in order to test the interstudy variability. Differences between the repeated measurements were evaluated by the paired Student's t-test. The coefficient of variability was expressed as the percentage difference for each pair of measurements as ((measurement observation 1 – measurement observation 2)/0.5 × (measurement observation 1+ measurement observation 2)) × 100 and expressed as mean ± SD. Another subset of 20 rats studies (10 rest and 10 stress) were analyzed by two independent observers, blinded for the results of each other, in order to test the interobserver variability. The interobserver variability in quantitative MCE was assessed by computing intraclass correlation coefficients using one-way ANOVA with a random factor and by calculating the limits of agreement between measurements made by use of Bland-Altman analysis [17]. A value of p < 0.05 was considered statistically significant. All statistical analyses were performed using SPSS (version 14.0) statistical software (SPSS Inc, Chicago, IL).

## Results

### Clinical parameters

Glucometry and gravimetric parameters are summarized in Table 1. After six months, animals treated with STZ had high glucose levels. Other symptoms frequently associated with diabetic state such as lower body weights, polyuria, polyphagia were observed in the diabetic rats. The heart mass decreased in the diabetic group but it was not significant compared to controls. The heart/body weight ratio significantly increased in the diabetic group.

**Table 1:** Glucometry and gravimetric data obtained at 6 months of diabetes

	**Glycemia (mg/dL)**	**Body mass (g)**	**Heart mass (mg)**	**Heart to Body Mass ratio (mg/g)**
Controls (n = 20)	95 ± 13	662 ± 49	1369 ± 222	2.1 ± 0.2
Diabetics (n = 20)	426 ± 54*	382 ± 34*	1182 ± 279	2.8 ± 0.3*

*p < 0.05

At the end of the follow-up there was no significant change in heart rate in the diabetic when compared to control rats nor in systolic blood pressure.

### Myocardial contrast echocardiography

Simple pulsing sequence during MCE was feasible in rats and it could assess myocardial blood velocity and volume separately, and myocardial blood flow as the product of the two. As shown in Table 2, during hyperaemia the basic haemodynamic parameters did not differ significantly in both groups. The myocardial blood flow estimate by MCE was also similar in controls and diabetics at rest and during hyperaemia. Interestingly, the β slope was significantly less steep in the control group compared to diabetics, both at rest and during hyperaemia. Although relative blood volume was not different at baseline in both groups, the rBV had significantly increased with hyperaemia in the controls compared to diabetics. Table 3 summarizes the changes in perfusion parameters and the myocardial perfusion reserve with hyperaemia. Although diabetics had a steeper β slope at rest and during hyperaemia, the change in β did not differ significantly between controls and diabetics. Conversely, changes in rBV were significantly more pronounced in the controls than in diabetics. As a result, the myocardial perfusion reserve was significantly higher in the control group.

**Table 2:** Haemodynamic and perfusion data at baseline and during hyperaemia in controls and diabetics.

	Baseline			Hyperemia		
		
Variable	Controls	Diabetics	p	Controls	Diabetics	p
Heart rate, min^-1^	318 ± 27	299 ± 33	NS	315 ± 29	316 ± 25	NS
SBP, mm Hg	131 ± 7	131 ± 13	NS	96 ± 3	100 ± 6	NS
RPP, min^-1 ^mm Hg	41,342 ± 1,234	39,056 ± 1,324	NS	31,339 ± 1,567	31,561 ± 1,234	NS
MBF, ml·min ^-1^·g ^-1^	2.42 ± 0.823	3.75 ± 0.943	NS	5.81 ± 0.99	5.02 ± 0.76	NS
β, min^-1^	23.3 ± 8.2	27.6 ± 9.1	†	32.3 ± 7.7	37.4 ± 9.9	†
rBV, ml·min ^-1^	0.112 ± 0.045	0.120 ± 0.05	NS	0.191 ± 0.046	0.141 ± 0.043	*

Values are mean ± SD. *p = 0.0001; †p = 0.005.

MBF = myocardial blood flow; rBV = relative blood volume;

RPP = rate-pressure product; SBP = systolic blood pressure.

**Table 3:** Changes in perfusion parameters and myocardial perfusion reserve with hyperaemia

	Changes with Hyperaemia		
	
Variable	Controls	Diabetics	p
MBF, ml·min^-1^·g ^-1^	3.09 ± 0.98	1.28 ± 0.67	†
β, min^-1^	9.1 ± 1.2	9.5 ± 1.1	0.06
rBV, ml·ml^-1^	0.073 ± 0.015	0.023 ± 0.009	*
MPR	2.39 ± 0.89	1.38 ± 0.43	†

Values are mean ± SD. *p = 0.0001;

†p = 0.005. MBF = myocardial blood flow; rBV = relative blood volume; MPR = myocardial perfusion reserve

Finally, no side effects due to contrast were observed and there was no significant change of the heart rate before and after contrast infusion both at rest and during hyperaemia.

### Interobserver and interstudy variability

**Figure 3:**
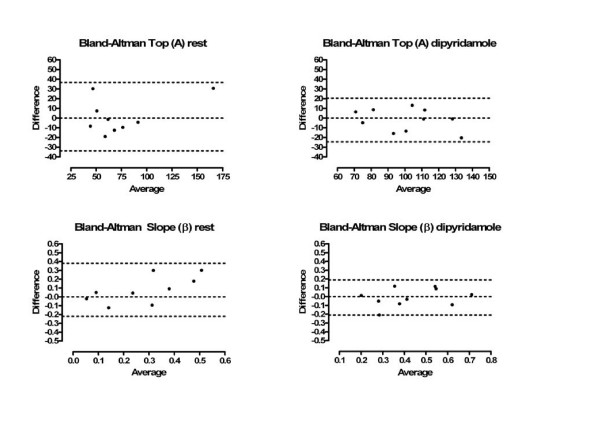
**Bland-Altman graphs for top (A) and slope (β) at rest and after dypiridamole in normal rats. **All measurements are within 2 SD of their differences demonstrating the agreement between the two independent observers.

Measurements were obtained in all rats. In the subset of 10 rats in which MCE was performed three days after the first examination in order to test the interstudy variability, differences between the repeated measurements were not statistically significant (p > 0.05). Interobserver variability was acceptable: 13.2 ± 10.1% and 14.5 ± 4.5% for top A and slope β, respectively. Bland-Altman analysis showed a good agreement between observers for both rest and DIP studies, as shown in the Figure 3.

### Echocardiographic measurements

Means and SD of the different anatomical parameters are given in Table 4. As can be derived from the M-Mode measurements, there was no difference between normal and diabetic rats after six months of follow-up both at rest and during DIP infusion, except for a decrease in inferior wall thickness at rest in diastole. When corrected for LV mass [18], a significant increase in LVESD (6.13 ± 1.8 vs 3.98 ± 0.03 mm/g; p < 0.001) and in LVEDD (13.98 ± 1.4 vs 5.12 ± 0.02 mm/g; p < 0.001) was observed in the diabetic group compared to controls.

**Table 4:** M-Mode parameters of the LV (parasternal short-axis view) measured in normal (n = 20) and diabetic rats (n = 20) after six months at rest and after dypiridamole infusion (DIP).

	Normal Rest	Diabetes Rest	Normal DIP	Diabetes DIP
Anterior wall diastole (cm)	0.21 ± 0.04	0.19 ± 0.04	0.21 ± 0.02	0.19 ± 0.02
Inferior wall diastole (cm)	0.21 ± 0.03	0.14 ± 0.03*	0.2 ± 0.02	0.18 ± 0.02
Anterior wall thickening (%)	49 ± 17	47 ± 15	58 ± 16	56 ± 16
Inferior wall thickening (%)	48 ± 16	46 ± 14	48 ± 12	49 ± 17
LV enddiastolic diameter (cm)	0.83 ± 0.09	0.88 ± 0.05	0.81 ± 0.07	0.85 ± 0.05
LV endsystolic diameter (cm)	0.47 ± 0.08	0.52 ± 0.05	0.48 ± 0.06	0.51 ± 0.05
LV enddiastolic volume (ml)	1.24 ± 0.3	1.49 ± 0.2	1.12 ± 0.3	1.30 ± 0.22
LV endsystolic volume (ml)	0.29 ± 0.12	0.34 ± 0.08	0.24 ± 0.08	0.28 ± 0.06
Fractional shortening (%)	41.42 ± 4.40	41.19 ± 3.92	40.4 ± 2.94	40.47 ± 3.48
LV ejectionfraction (%)	75.22 ± 4.93	74.86 ± 4.21	78.69 ± 3.01	78.71 ± 3.41
Left ventricular mass (g)	1.35 ± 0.21	1.22 ± 0.28	1.34 ± 0.17	1.23 ± 0.22

* indicates p < 0.05

### Histology

There was no difference regarding extra-cellular collagen deposits (trichrome staining) or endomyocardial necrosis (heamatoxilin-eosin staining) in myocardium of control and diabetic rats at the end of the follow up. The percentage of fibrosis was less than 1%, with no difference between the two groups at the end of the follow up. Mean capillary surface area (16.2 ± 1.7%, p = 0.01) and capillary diameter (11.9 ± 0.5 μm, p = 0.04) were significantly lower in the diabetic animals compared to the controls (11.0 ± 1.2% and 10.8 ± 0.3 μm, respectively). No microangiopathy involving arterioles, capillaries, venules, and hyaline arteriosclerosis was observed.

## Discussion

The study of cardiomyopathies in small animals may contribute to our understanding of the cardiac pathophysiology and to evaluate experimental treatment strategies. The present study shows that myocardial perfusion imaging with contrast echocardiography can successfully be applied for the non-invasive evaluation of cardiac perfusion at rest and under hyperaemia in STZ-induced diabetic rats. Our results suggest that after six months, diabetes induces a functional alteration of the myocardial microcirculation that may explain left ventricular systolic dysfunction. Our data further indicate that microcirculation is already altered at rest and that the major determinant for decreasing myocardial perfusion reserve during hyperaemia, is the lower capillary recruitment in the diabetic group. Finally, histopathology findings demonstrate a reduced density of myocardial capillaries, in the diabetic group that can only be unmasked with hyperaemia using MCE.

### Clinical parameters

The STZ induced diabetic rats used in our experiments are reminiscent of a model of uncontrolled hyperglycemia due to the direct pancreatic beta cell destruction and resulting insulin deficiency [19]. Our glucometry and gravimetric values (Table 1) are in accord with other published values at similar time points [20-22]. These STZ rats resembled more type 1 diabetes. The dose of STZ injected (45 mg/kg) was low in this study. However, it has been successfully applied in previous studies [3,14] and the impact on glucometry and gravimetric values was similar compared to the administration of a higher dose of STZ, leading to equivalent absolute insulin deficiency.

### Myocardial perfusion

Microbubbles are excellent tracers of red blood cell kinetics. They are pure intravascular tracers. The method to quantify MBF is based on rapid destruction of these microbubbles by ultrasound, and subsequent assessment of the rate of replenishment into the myocardium within the ultrasound beam elevation. This method allows the assessment of the components of MBF: flow velocity and myocardial blood volume [13]. In the presence of increased myocardial oxygen demand, there is arteriolar vasodilation, reducing the resistance at the arteriolar level, which enables a higher precapillary pressure, translating into increased red blood cell velocity across the capillary network, and opening dormant capillary networks in order to maintain mean trans-capillary pressure (recruitment), thus increasing the overall myocardial blood volume. Hence overall myocardial blood flow is increased.

The present study has shown that quantitative MCE is a robust method to assess the myocardial blood flow in small animals at rest and during hyperemia. The measurements are feasible and repeatable over time and between observers. As expected from physiology and previous in vitro and in vivo studies [9,10,13,23,24], we were able to show the increase of MBF with hyperemia in normal rats. Moreover, our data were consistent with previous experiments in small animals, validating MCE measurements using microspheres [15].

In our diabetic population, we could observe, at rest, a significant increase in β compared to controls. This suggests a decrease in arteriolar resistance and vasodilation of arterioles. Although this increase in red cell velocity at rest compared to controls persists under hyperaemia in the diabetics versus the controls, the change in β is not significantly different in diabetics compared to controls. This finding suggests the capacity of arterioles to further vasodilate, even in the diabetic group, in the absence of coronary artery lesions.

Structural alterations of the small vessels in diabetes have been incriminated in the development of diabetic cardiomyopathy, although this remains controversial. A reduction in capillary density and a significantly greater thickening of the capillary basement membrane has been shown compared to control subject in humans [25] and more recently in rodents [26]. Because rBV represents essentially capillaries, one would therefore expect to find a decrease in A in the diabetic rats, which is not the case at rest. However, during hyperaemia, there is less increase of rBV in diabetic rats compared to normal rats, indicating a less important capillary recruitment in diabetics. This may reflect the absence of compensation to an increased pre-capillary pressure mediated by arteriolar vasodilatation, as shown by the increased red cell velocity at rest and during hyperaemia compared to controls. In our study, histopathology did confirm a significant reduction in capillary density, in accordance with the previous studies [25,26]. The increase in pre-capillary pressure induced by the arteriolar vasodilation and the decrease of capillary density may be compensated by a capillary recruitment at rest in the diabetic group resulting in no significant change of rBV. We can hypothesize that hyperaemia is required to unmask the decrease of capillary density in diabetics, using MCE. As a net result, myocardial perfusion reserve is altered and may lead to relative ischemia. Similarly, a reduction of myocardial blood flow and significant increase in total coronary resistance during hyperaemia and consequent impairment of coronary flow reserve have been reported in type I young adult diabetic patients with no or minimal microvascular complications and without any evidence of coronary heart disease [27]. Reduced myocardial flow reserve may lower the threshold for myocardial ischemia, particularly when coronary stenoses are present. It has been proposed that diabetic cardiomyopathy is a consequence of repeated episodes of myocardial ischemia resulting from these functional abnormalities in small vessels during increased myocardial demand. As shown by Litwin and colleagues, a real insulin-therapy that aims to normalize the glycemia, also corrects the cardiac abnormalities [28]. An ongoing study investigates the effect of the correction of diabetes condition on the impairment of coronary circulation.

### Left ventricular function

There remains controversy regarding diabetes-induced LV dysfunction, especially in type 1 diabetes, in the absence of documented coronary artery disease. Some authors have been able to detect early systolic LV dysfunction and dilatation of the left ventricle in STZ induced diabetic cardiomyopathy [29,30]. On the contrary, others were not able to demonstrate a remodelling and a significant alteration of the systolic function in a similar rat population [31]. A recent study in patients with type 1 diabetes, even with the application of echocardiography, biochemical and morphologic techniques, failed to demonstrate that diabetes type 1 may actually precipitate myocardial dysfunction and no heart-specific, histological changes in the myocardium were found. However, as acknowledged by the authors of this latter study, all the patients were treated with intensive insulin therapy [32].

Standard measurements of LV wall thickness and systolic and diastolic LV diameters by M-mode have extensively been described in normal and diseased rat models [33-36]. The ratio data such as fractional shortening and ejection fraction have been demonstrated to be similar in rat and human echocardiography [36]. Comparable results were obtained in the present study. In literature anatomical M-Mode and bi-dimensional echocardiography have been able to detect early systolic LV dysfunction and dilatation of the left ventricle in STZ induced diabetic cardiomyopathy [22,29,37-39]. We observed an increase in mean normalized EDV of the diabetic group compared to controls. Meanwhile the normalized ESV of the diabetic rats also increased compared to controls but in lower proportion. The significant increase of normalized EDV with diabetes in this study is in accordance with previous reports [22,29,40,41]. However, in contrast to our findings, other authors have shown no significant change or even an decrease in this parameter [42-44]. We also observed an increase in normalized ESV, suggesting a decrease in contractility, in accord with all previous studies [22,29,40,43,44]. The differences in LV volumes may be due to the method used to normalize the data or to theabsence of normalization to body weight in other studies. Allometric relations exist between cardiac and body size measurements. However, the correct method to use in rat is unknown. We normalized LV mass to bodyweight as applied in the previous studies using the same animal model [22,41]. In addition, these differences may reflect the difference in the strain of rats (Wistar Unilever, Wistar Kyoto, Sprague-Dawley), since this factor has been shown to clearly influence cardiomyopathy in the STZ model of diabetes [45].

### Histopathology

In the present study, no significant pathological changes were observed in diabetic rats, regarding extra-cellular collagen deposits, endomyocardial necrosis in myocardium, and no microangiopathy involving arterioles, capillaries, venules, and hyaline arteriosclerosis was present, at the end of the follow up. Conversely, in our study, histopathology did confirm a significant reduction in capillary density. These results are in accordance with previously published works [25,26]. The muscular fibers were thinner in the diabetic rats: 14.6 ± 3.1 μm in controls and 11.4 ± 2.8 μm in diabetics (P = 0.043). This last finding was also consistent with previously published work (23).

## Limitations

Our STZ model resembled more type 1 diabetes and therefore, our results may not be applied to other forms of diabetes. Using M-Mode, we have only performed radial measurements of diameters and therefore volumes were calculated based on these measurements. However, it has been previously shown that M-mode echocardiographic measurements are representative of ventricular function and volumes measurements, compared to other imaging techniques [3,43]. No significant structural abnormalities were noted in the myocardium after 6 months. However, electron microscopic was not performed in the present study and previous studies using this method have repeatedly documented that diabetes is indeed associated with abnormalities in myocardial structure [46]. This may partially explain the left ventricular dysfunction despite the absence of abnormal findings at histology in our study.

## Conclusion

Myocardial perfusion imaging with contrast echocardiography can successfully be applied for the non-invasive evaluation of cardiac perfusion at rest and under hyperaemia in STZ-induced diabetic rats. After six months, diabetes induces a functional alteration of the myocardial microcirculation and a left ventricular systolic dysfunction. Microcirculation is already altered at rest. The major determinant for decreasing myocardial perfusion reserve during hyperemia, is the lower capillary recruitment in the diabetic group. Histopathology findings demonstrate a reduced density of myocardial capillaries in the diabetic group that can only be unmasked with hyperaemia using MCE. These findings may offer new insights into the underlying mechanisms of diabetes cardiomyopathy.

## Competing interests

The authors declare that they have no competing interests.
